# Radiological perspective on earthquake trauma: differences in children and adults

**DOI:** 10.1590/1806-9282.20240683

**Published:** 2024-11-11

**Authors:** Gökhan Mert Özyurt, Sarhun Zirek

**Affiliations:** 1 Mersin City Hospital, Department of Radiology – Mersin, Turkey.

**Keywords:** Earthquake, Injury, Children, Adults

## Abstract

**OBJECTIVE:**

February 2023 saw major earthquakes in Pazarcık and Elbistan, causing significant devastation in Turkey. Patients were transferred to hospitals in neighboring provinces, with multiple traumas—especially fractures and organ injuries—forming the main reasons for hospital admissions. This study aimed to examine earthquake-related injuries in pediatric and adult populations to understand differences.

**METHODS:**

This study analyzed 1,220 adults and 590 pediatric patients with radiological imaging out of 8,704 earthquake trauma cases. Radiological images were assessed independently by two radiologists. Statistical analysis using SPSS examined relationships between variables such as age group and injury type.

**RESULTS:**

Results showed 40% of adults and 64% of children had normal radiological findings. Cerebral and extremity traumas were most common in pediatrics, while adults showed more extremity, thoracic, and spinal traumas. Significant differences between adult and pediatric groups were observed in cranial fractures, thoracic and lumbar vertebral fractures, hemopneumothorax, lung contusions, rib fractures, femur and talocalcaneal fractures, and compartment syndrome (p<0.001).

**CONCLUSION:**

Earthquake-related injuries may vary between children and adults. Due to children's more flexible anatomical structure, it is believed that earthquake-related injuries occur less frequently in children. In this study, head traumas were more common in children compared to adults. The rate of cranial fractures was significantly higher in children, with a higher incidence of epidural hematoma compared to adults. Spinal traumas were more frequent in adults than in children, attributed to children's greater flexibility reducing the risk of entrapment under rubble. Pediatric thoracic compliance being significantly higher than in adults often resulted in milder chest traumas. However, compartment syndrome was more common in children, with a lower rate of accompanying bone fractures compared to adults. No significant difference was observed between children and adults in maxillofacial, abdominal, and pelvic traumas. These findings provide insights for future disaster healthcare planning and management.

## INTRODUCTION

In February 2023, two separate earthquakes of magnitudes 7.8 Mw and 7.5 Mw struck the districts of Pazarcık and Elbistan, respectively^1^. These earthquakes caused tremors of up to maximum intensity XII (disastrous) on the Mercalli intensity scale. According to official records in Turkey, at least 53,537 people lost their lives, and more than 122,000 were injured as a result of these earthquakes. Following the earthquakes, more than 45,000 aftershocks, with magnitudes of up to 6.7 Mw, occurred^2^. Due to the complete collapse of infrastructure and healthcare services in the earthquake-affected areas, patients were transferred to hospitals in neighboring provinces.

Multiple traumas, such as bone fractures, soft tissue injuries, and organ damage caused by building collapses or falling objects, constitute the most common reasons for hospital admissions after a major earthquake^3^. Rapid and reliable detection of the clinical condition in trauma cases is crucial, and radiological imaging plays a vital role in this regard. Next-generation computed tomography (CT) scans with multiplanar reconstruction are highly valuable diagnostic tools for detecting head, chest, spine, abdominal, and pelvic injuries^4^. Additionally, ultrasound (USG) in patient triage and direct radiography for detecting limb fractures are also essential^5^.

Strong disaster preparedness is essential to provide optimized intervention in a chaotic environment immediately following an earthquake. Radiologists and clinicians need to be knowledgeable and prepared for earthquake-related traumas. This article aimed to elucidate the radiological findings and trauma patterns related to earthquake-induced injuries in pediatric and adult populations and to identify potential differences and similarities by comparing these findings with those reported in the literature.

## METHODS

This study was conducted in accordance with the Helsinki Declaration and was approved by the Mersin University Clinical Research Ethics Committee. Due to the retrospective nature of the study design, informed consent was waived. A total of 8,704 individuals presented to the hospital due to earthquake-related trauma, out of which 1,220 adults and 590 pediatric patients with a history of being trapped under rubble and undergoing radiological imaging were included in the study. Close to 10,000 CT scans and around 20,000 direct radiographs were reviewed. In total, 53 adult and 46 pediatric patients were excluded from the study due to inadequate image quality. Radiological images stored in the hospital's PACS system were independently evaluated by two radiologists, and any discrepancies were resolved. Patients were categorized into seven groups based on the type of injury: cerebral, spinal, maxillofacial, thoracic, abdominal, pelvic, and extremity injuries. Patients aged 0–18 years were classified as pediatric, while those above 18 years were classified as adults.

**Figure 1:**
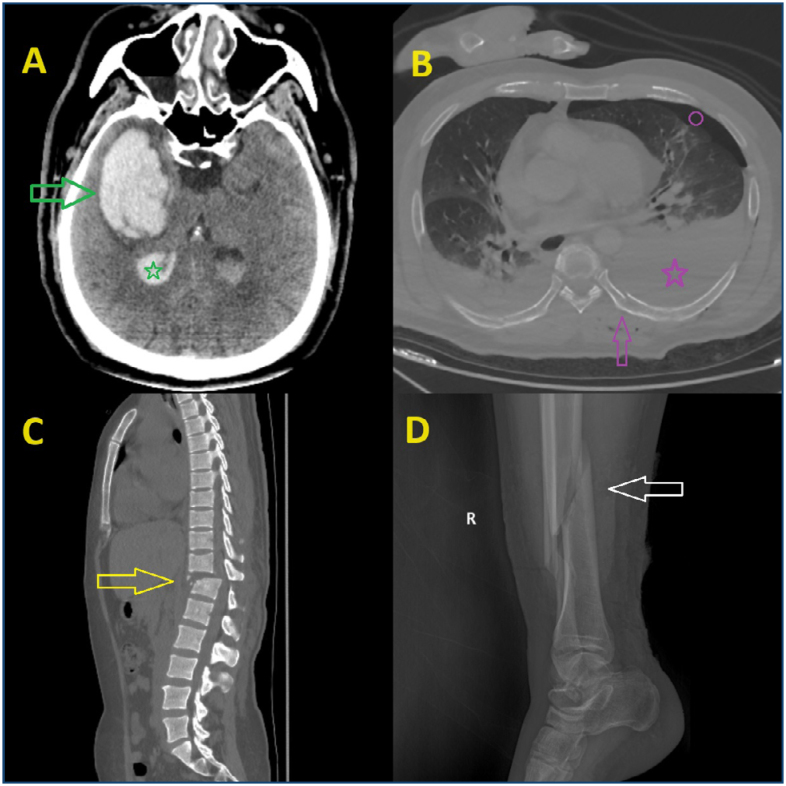
Several common types of injuries observed in earthquake-induced trauma are illustrated. In the patient with cerebral trauma **(A)**, a parenchymal hematoma (green arrow) and intraventricular hemorrhage (green star) are shown in the right temporal lobe. In the patient with thoracic trauma **(B)**, a posterior fracture of the left seventh rib (purple arrow), hemothorax (purple star), and pneumothorax (purple circle) are demonstrated. In the patient with thoracolumbar trauma **(C)**, a vertebral fracture with posterior displacement and translational-rotational type at the T12 vertebra level is observed (yellow arrow). In the patient with trauma to the right lower leg **(D)**, a comminuted fracture in the distal tibia and fibula is noted (white arrow).

Patients participating in our study were rescued from under the rubble through individual efforts or the efforts of rescue teams from the first hours after the earthquake and throughout approximately 1 week. They were then transported to our hospital by sea, air, or road. All patients trapped under the rubble, regardless of the degree of trauma, underwent whole-body CT imaging (cerebral, spinal, thoracic, abdominal, and pelvic) and direct radiographic imaging of the upper and lower extremities. Extremity fractures were identified using direct radiographs, and in cases of suspected fractures and for patients scheduled for surgery, extremity CT imaging was performed. Cerebral, spinal, thoracic, and abdominal injuries were evaluated using CT (Figure 1). Due to the history of widespread crush injuries, the use of contrast agents was minimized as much as possible. Patients who underwent fasciotomy surgery based on clinical history and physical examination findings indicative of compartment syndrome were classified as having compartment syndrome. Magnetic resonance imaging findings were not included in the study.

A significant limitation of this study is the lack of detailed data on the patient's clinical conditions, injury severity (e.g., Glasgow Coma Score), duration of entrapment, length of hospital stay, and survival status. This limitation imposes restrictions on the findings of our study. Future research should aim to collect and analyze such detailed data to address these limitations.

The patients’ data were entered into a Microsoft Excel spreadsheet. Subsequently, statistical analyses of these data were conducted using SPSS (IBM SPSS Statistics 29.0.2.0). For statistical analysis, the chi-square test was employed. This test was used as an independent test to evaluate the relationship between two categorical variables. The independent categorical variables were age group and type of injury.

## RESULTS

**Figure 2:**
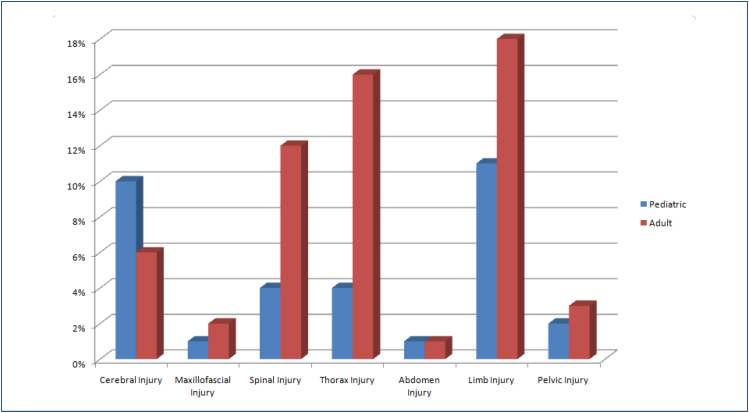
Clustered bar graphs show the distribution percentages of earthquake-related injuries among adult and pediatric populations.

A total of 1,810 patients who were trapped under rubble during the earthquake centered in Kahramanmaraş on February 6, 2023, were evaluated radiologically in this study. Among these patients, 714 were female and 506 were male. Of the pediatric patients, 282 were male and 308 were female. The mean age of the participants was 46.3 years for adults and 11.1 years for children. The youngest participant was 8 months old, while the oldest was 86 years old. Radiological findings were entirely normal in 497 adult patients and 381 pediatric patients. The proportion of patients with normal radiological findings was 40% in adults and 64% in children. The most common traumas in the pediatric population were cerebral and extremity injuries, while in adults, they were extremity, thoracic, and spinal injuries (Figure 2).

Significant differences were observed between adult and pediatric groups in cranial fractures, thoracic and lumbar vertebral fractures, hemothorax, pneumothorax, lung contusion, rib fractures, femur fractures, and talocalcaneal fractures. There was also a significant difference in compartment syndrome (Table 1).

**Table 1:** Distribution of anatomical regions affected by earthquake-related trauma in adult and pediatric groups.

Trauma types	Pediatric	Adult	Total	p
N	590	1,220	1,810	
Cerebral trauma	124	146	270	0.001
Spinal trauma	48	296	344	0.001
Maxillofascial trauma	18	46	64	0.671
Thorax trauma	54	402	456	0.001
Abdominal trauma	10	30	40	0.517
Pelvic trauma	28	72	100	0.578
Limb trauma	134	436	570	0.001

None of the patients included in the study had biliary or intestinal injuries. Injuries to the bladder, uterus, ovaries, and testicles were also not observed.

## DISCUSSION

Earthquake-related injuries may vary between children and adults. The structure and anatomy of children are more flexible and elastic compared to adults. This difference may lead to fewer earthquake-related injuries occurring in children. Injury mechanisms during earthquakes can vary; children are generally more prone to falls, whereas adults may encounter risks such as being crushed by larger objects or being trapped under rubble^6^.

One notable feature of this study is the higher incidence of head traumas in children compared to adults. While the rate of head trauma in adults was 6%, it was determined to be 10% in children. A significant difference was observed between adult and pediatric populations in cerebral traumas (p<0.001). Studies in the literature have reported varying rates; Farfel et al. reported head trauma in 3.2% of 155 cases, whereas Zhao et al. indicated that 12% of 192 cases had head trauma^7,8^. Children have relatively larger and heavier heads compared to their bodies, supported by weaker muscles and ligaments, making them more susceptible to injury compared to adults^9^. Although children's bones are more flexible, the calvarium is thinner, making it more susceptible to deformation under external pressure^10^. This study revealed a significant difference in cranial fractures between children and adults (p<0.001), with a higher incidence in children. The middle meningeal artery in children is not as integrated into the bone as in adults, making epidural hematomas more likely to occur^11^. The rate of epidural hematoma was higher in the pediatric population in this study (Table 2).

**Table 2:** Distribution of trauma types related to the earthquake in adult and pediatric groups.

Trauma types	Pediatric	Adult	Total	P
N	590	1,220	1,810	
Subarachnoidal hemorrhage	10	24	34	0.851
Epidural hemorrhage	8	4	12	0.077
Subdural hemorrhage	20	36	56	0.719
Cerebral contusion	16	24	40	0.419
Cranium fracture	50	30	80	0.001
Skull base fracture	6	4	10	0.189
Maxillofascial fracture	18	46	64	0.671
Cerebral edema	12	14	26	0.268
Brain herniation	12	10	22	0.405
Cervical vertebra fracture	4	14	18	0.534
Thoracic vertebra fracture	18	94	112	0.005
Lomber vertebra fracture	18	148	166	0.001
Sacrum fracture	8	40	48	0.093
Hemothorax	14	82	96	0.006
Pneumothorax	6	66	72	0.001
Lung contusion	12	110	122	0.001
Rib fracture	14	110	124	0.001
Pneumomediastinum	8	30	38	0.304
Hemopericardium	0	4	4	0.218
Liver injury	4	10	14	0.865
Pancreas injury	0	2	2	0.384
Biliary injury	0	0	0	0
Splenic injury	0	6	6	0.131
Intestinal injury	0	0	0	0
Renal injury	6	12	18	0.907
Shoulder dislocation	0	6	6	0.131
Humerus fracture	12	36	48	0.477
Clavicula fracture	6	26	32	0.245
Radioulnar fracture	8	26	34	0.463
Carpal bone fracture	6	14	20	0.917
Pelvic dislocation	2	16	18	0.147
Femur fracture	18	110	128	0.001
Patella fracture	2	12	14	0.291
Tibiofibular fracture	14	62	76	0.063
Talocalcaneal fracture	2	44	46	0.001
Iliac fracture	12	22	34	0.738
Pubic fracture	16	50	66	0.411
Vascular injury	2	18	20	0.104
Compartment syndrome	64	84	148	0.027

In spinal traumas, a significant difference was observed between adults and children (p<0.001). While 12% of adult patients had spinal trauma, this rate was 4% in children. Traumas in the spinal region were mostly in the form of thoracic and lumbar vertebral fractures, with fewer cervical injuries. Transverse process fractures and compression fractures were most common. Studies indicate that individuals trapped under debris during earthquakes often fall and become trapped in a fetal position^12^. Children are generally more flexible and agile, making it easier for them to escape tight spaces and react more quickly. Due to their smaller size, they are at lower risk of being trapped under debris. Therefore, it can be said that children have an advantage in terms of spinal injuries compared to adults (Table 1).

In this study, thoracic injuries were found to be 16% in adults and 4% in children, with a statistically significant difference (p<0.001). Rib fractures and lung contusions were the most common types of injuries. No tracheal, diaphragmatic, or esophageal injuries were observed in the screened patients. Following blunt trauma, the likelihood of rib fractures in pediatric patients is lower compared to adults due to the elastic nature of their ribs, and such fractures require a higher amount of energy. Therefore, isolated rib fractures are a more common clinical presentation in adults, whereas, in pediatric patients, rib fractures are often accompanied by conditions such as pneumothorax, hemothorax, or lung contusion^4,13^. In a study by Kessel and colleagues, the rate of isolated rib fractures was reported to be 11% in adults and 5.8% in children. The study also found that associated brain injury (p=0.003), hemothorax/pneumothorax (p=0.006), and spleen and liver injuries (p<0.001) were more prevalent in children^13^. In our study, differing from the literature, rib fractures (p<0.001), hemothorax (p=0.006), pneumothorax (p<0.001), and lung contusion (p<0.001) were more frequently observed in adult patients (Table 2). We believe the reason for this is the much higher energy transfer in earthquake-related trauma compared to routine blunt thoracic trauma.

Compartment syndrome is a clinical diagnosis, and the role of radiological imaging is limited. Imaging findings typically manifest as edema and swelling in muscle tissues. If the increase in compartment pressure due to edema exceeds perfusion pressure, ischemic symptoms begin, leading to painful myonecrosis and nerve damage^14^. Studies have suggested that compartment syndrome is ten times more common in men and more frequent in individuals under 35 years old^15^. In our study, the frequency of compartment syndrome was higher in the pediatric population, with a significant difference between adult and pediatric populations (p=0.027) (Table 2). Among pediatric patients with compartment syndrome, 62% had an accompanying bone fracture, while this rate was 86% in adults. Tibiofibular fractures were most common in children with compartment syndrome, while femur fractures were most common in adults.

There were no significant differences between adult and pediatric patients in terms of maxillofacial, abdominal, and pelvic traumas (p=0.671, p=0.517, and p=0.578, respectively).

This study has several limitations. Due to the chaos following the earthquake, clinical history and physical examination findings were not adequately documented for some patients. Consequently, aspects such as the duration of time patients remained under debris, trauma scores, and similar characteristics could not be assessed within the scope of this study. Additionally, the absence of magnetic resonance imaging evaluations resulted in the inability to assess spinal cord damage, which constitutes another limitation.

## CONCLUSION

This study conducted a comprehensive analysis to categorize patients based on their radiological findings and types of trauma. The results have revealed significant differences in earthquake-related injuries between children and adults. Particularly, it was found that head traumas are more common in children compared to adults. Additionally, a higher incidence of compartment syndrome cases was observed in the pediatric population, while thoracic traumas were less frequent in children. In the discussion section, the underlying reasons for these differences and measures to be considered in managing post-disaster healthcare services were addressed. The findings of this study can serve as an important guide for planning and managing healthcare services in future disasters.
